# Case Report: Murine typhus complicated by symmetrical peripheral gangrene: first report and diagnostic insights from metagenomic next-generation sequencing

**DOI:** 10.3389/fimmu.2025.1746919

**Published:** 2026-01-16

**Authors:** Hengling Zhu, Linhui Hu, Zaiming Feng, Zichen Zhang, Hengbao Zhu, Huihua Li

**Affiliations:** 1 Department of Critical Care Medicine, Maoming People’s Hospital, Maoming, Guangdong, China; 2 The First School of Clinical Medicine, Southern Medical University, Guangzhou, Guangdong, China; 3 The First School of Clinical Medicine, Guangdong Medical University, Zhanjiang, Guangdong, China; 4 Children’s Hospital, Zhejiang University School of Medicine, Hangzhou, Zhejiang, China; 5 Department of Pharmacy, Maoming People’s Hospital, Maoming, Guangdong, China

**Keywords:** metagenomic next-generation sequencing (mNGS), multiple organ dysfunctions (MODS), murine typhus, *Rickettsia typhi* (*R. typhi*), symmetrical peripheral gangrene (SPG)

## Abstract

**Background:**

Murine typhus, a flea-borne infection caused by *Rickettsia typhi*, often presents with nonspecific symptoms that delay diagnosis. While usually self-limiting, it can rarely progress to multiple organ dysfunction syndrome (MODS). We report the first case of murine typhus complicated by symmetrical peripheral gangrene (SPG), in which metagenomic next-generation sequencing (mNGS) enabled rapid diagnosis and guided timely doxycycline therapy.

**Case presentation:**

A 69-year-old female from South China was hospitalized with persistent abdominal pain and low-grade fever. She was a farmer and had suspected animal exposure. Laboratory investigations revealed hypoxia, abnormal coagulation profile, hepatorenal impairment, and thrombocytopenia. Despite empirical antibiotic therapy, her condition deteriorated progressively, manifested as hemodynamic instability, respiratory failure, and the emergence of purpuric-petechial cutaneous eruptions. Immediate interventions were initiated, including administration of vasoactive agents and mechanical ventilation. Based on mNGS, *R. typhi* was confirmed, she received targeted antibiotic treatment with intravenous doxycycline (100 mg twice daily). On the hospital day 16, gangrene of all four extremities became evident. The patient underwent amputation of all four extremities and survived, with systemic symptoms gradually resolving during 6-months follow-up.

**Conclusion:**

This first reported case of murine typhus complicated by symmetrical peripheral gangrene (SPG) establishes its potential to cause life-threatening multiorgan failure. Metagenomic next-generation sequencing (mNGS) resolved the diagnostic challenge by rapidly identifying *Rickettsia typhi*, guiding life-saving doxycycline therapy and underscoring its value in severe zoonotic infections.

## Background

Murine typhus, a flea-borne zoonosis caused by *Rickettsia typhi (R. typhi)* and primarily transmitted by *Xenopsylla cheopis*, is widely distributed in warm and humid regions such as Southeast Asia, the Mediterranean basin, and the southern United States (1–4). Clinically, its classic triad of fever, headache, and rash occurs in only one-third of patients, while other nonspecific symptoms—such as malaise, myalgia, and gastrointestinal discomfort—often delay diagnosis (4). The disease is maintained in nature through reservoirs including opossums, rats, and household pets, with seasonal transmission peaks corresponding to flea proliferation (5, 6). In China, although competent vectors are prevalent, particularly in subtropical regions like Guangdong Province, reported human cases and clinical data on severe manifestations remain limited (4, 7). A systematic review found that 26.1% of patients develop complications such as pneumonia, acute renal failure, or altered mental status, highlighting its underestimated clinical burden (4).

Despite this, critical knowledge gaps persist. Most reports describe mild or moderate infections, while cases progressing to multiorgan dysfunction syndrome (MODS) are exceedingly rare—only 15 such cases have been documented worldwide, with a mortality rate exceeding 50% (8). Moreover, symmetrical peripheral gangrene (SPG), a severe ischemic complication typically associated with meningococcal sepsis or disseminated intravascular coagulation, has never been linked to murine typhus (9–11). Diagnostic delays further compound clinical risk, as conventional serology is slow, polymerase chain reaction (PCR) has limited sensitivity, and metagenomic next-generation sequencing (mNGS)—a promising tool for identifying rare pathogens—has not been evaluated in this setting (8, 12).

Here, we report the first documented case of murine typhus complicated by SPG, confirmed by mNGS in a critically ill patient with MODS who survived following targeted doxycycline therapy and multiple amputations. To contextualize this unprecedented presentation and delineate the clinical spectrum of severe murine typhus, we additionally performed a systematic review of all published *R. typhi*-associated MODS cases worldwide. By integrating this rare case with existing literature, our study aims to bridge current evidence gaps, highlight diagnostic advances offered by mNGS, and provide practical guidance for the management of atypically severe rickettsial infections in endemic regions.

## Case presentation

**Figure 1:**
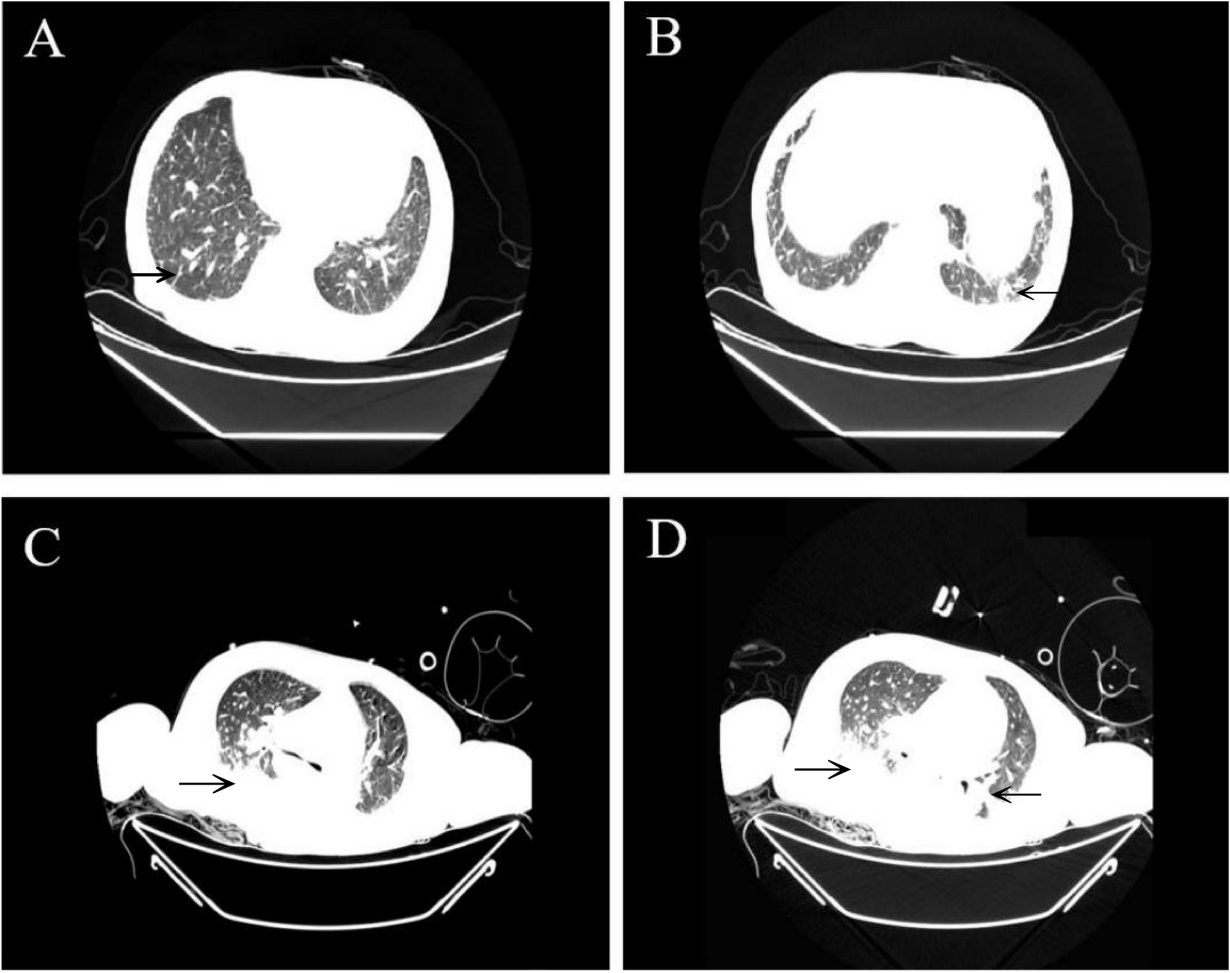
Chest CT scans during hospitalization and follow-up. Axial chest CT images obtained on the first **(A, B)** and sixth **(C, D)** days after admission. Black arrows depict progressive bilateral lower-lobe infiltrates and increasing pleural effusion.

A 69−year−old female farmer, who had prolonged rural residency, presented to the emergency department with 2-days progressive abdominal pain and 24-hours low-grade fever (38.2 °C). She had a history of hypertension without other significant comorbidities. Recent fieldwork exposure was noted, with absence of maculopapular rash, eschar, or hemorrhagic manifestations. Initial laboratory findings revealed mild leukocytosis (14 ×10^9^/L, 89% neutrophils), thrombocytopenia (51×10^9^/L), elevated C-reactive protein (68.53 mg/L), and procalcitonin (1.63 ng/mL). To rule out other major endemic infections, we performed multiple diagnostic tests: the Widal test for typhoid and paratyphoid was negative (all titers <1:80), and scrub typhus was excluded by a consistently negative Weil-Felix OXK titer (<1:80). Furthermore, nucleic acid testing for SARS-CoV-2 was negative. To differentiate the symmetrical peripheral gangrene from non-infectious causes, we conducted screening for systemic vasculitis; both the anti-neutrophil cytoplasmic antibody (ANCA) profile (including MPO, PR3, and GBM antibodies) and the comprehensive anti-nuclear antibody (ANA) profile were negative, effectively ruling out common autoimmune-mediated vasculitides. Thoracic computed tomography (CT) demonstrated bilateral lower lobe patchy opacities with small amounts of pleural effusion (**Figures 1A, B**). Abdominal CT showed pancreatic duct dilation, a small hepatic cyst, with unremarkable gallbladder, spleen, adrenal glands, bladder, and adnexa. Following a presumptive diagnosis of biliary tract infection, she received empirical antimicrobial therapy of imipenem and morinidazole.

By day 3, the patient exhibited clinical deterioration by high fever, lethargy, myalgia, and tachypnea. On day 4, progressive respiratory failure developed with critical hypoxemia (SpO_2_ 80%) and hemodynamic instability accompanied by purpuric and petechial rash. Repeat CT revealed extensive bilateral pulmonary infiltrates and increased pleural effusion, indicating severe pneumonia progression (**Figures 1C, D**). Due to persistent hypoxemia and hypotension, she was transferred to the intensive care unit (ICU) where she received mechanical ventilation and inotropic support. Her lab work showed rising liver-associated enzymes (alanine aminotransferase 498.6U/L), hyperbilirubinemia, acute kidney injury (creatinine of 99.49umol/L), coagulation disorder (PT 20.6 s, PTT 52.1 s), and elevated cardiac biomarkers (myoglobin 299 ng/mL, troponin T 209 ng/L, and BNP 17,314.6pg/mL). Bedside bronchoscopy demonstrated diffuse mucosal inflammation without purulent secretions, masses, or active bleeding. Bronchoalveolar lavage (BAL) specimens returned negative for bacterial cultures, fungal elements, and acid-fast bacilli.

**Figure 2:**
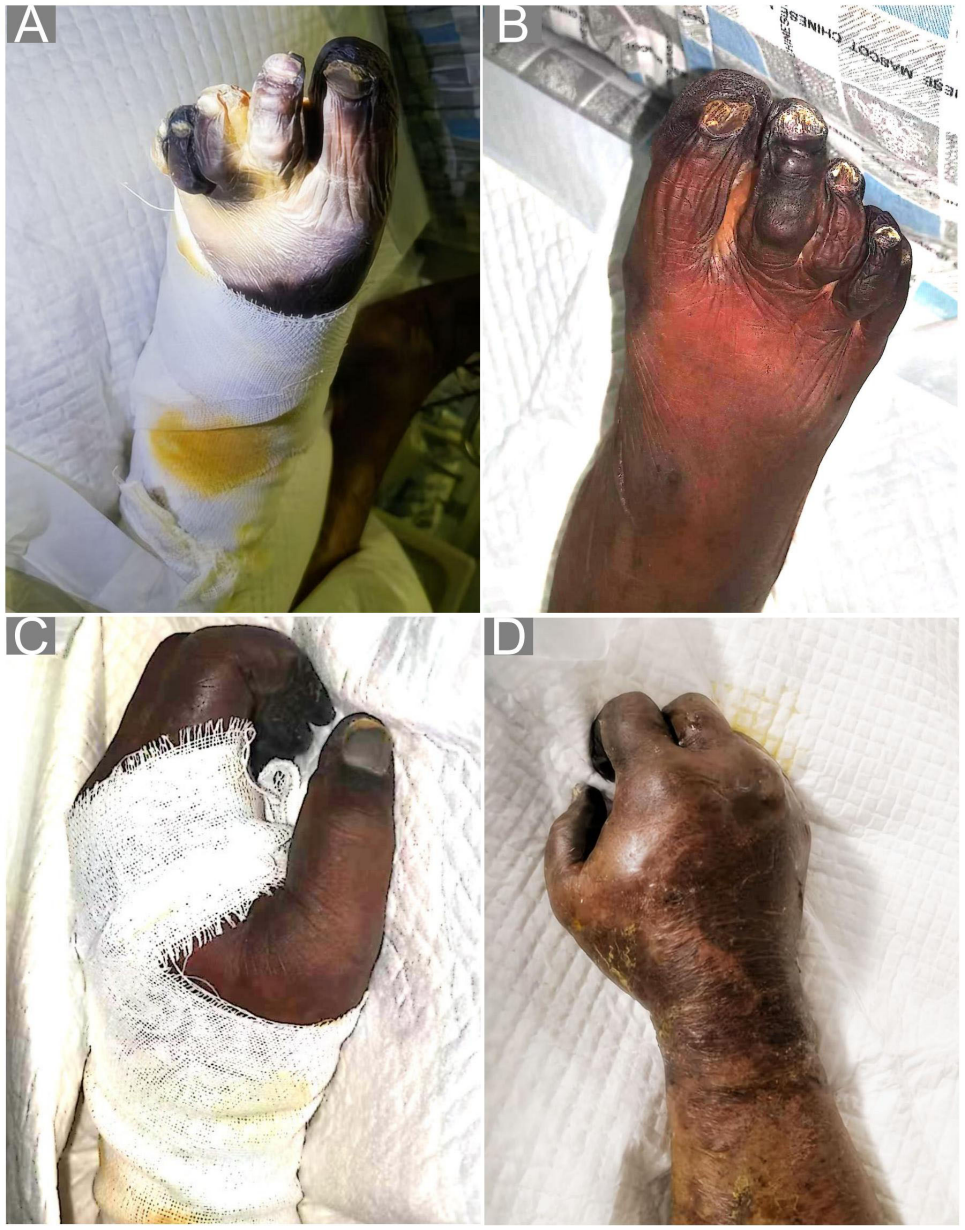
Well-demarcated dry gangrene affecting all four limbs on day 16. Clinical photographs taken on day 16 demonstrating symmetrical dry gangrene of both lower **(A, B)** and upper **(C, D)** extremities, with clearly demarcated areas of tissue necrosis.

**Figure 3:**
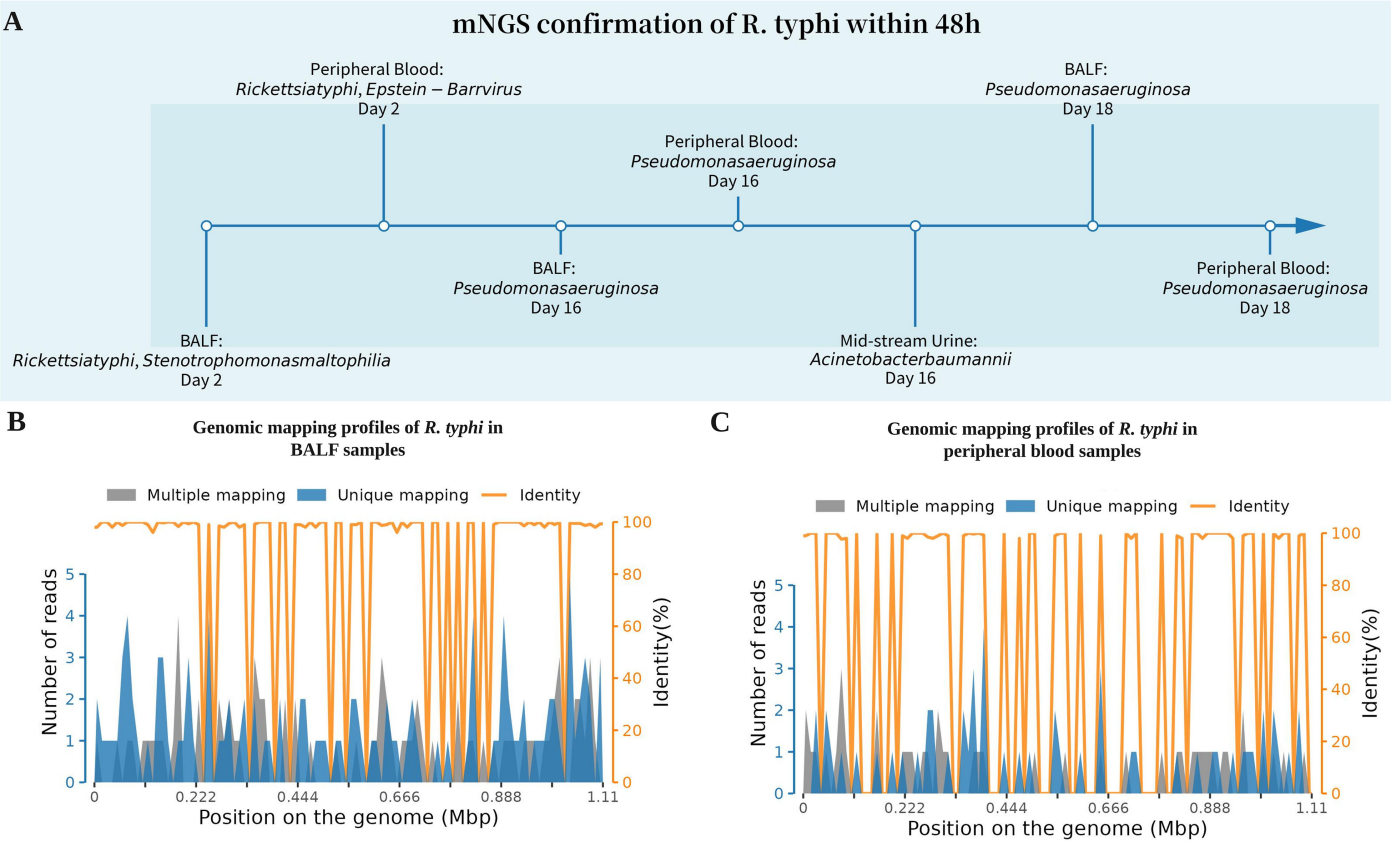
Detection of R. typhi **(A)** Timeline of pathogen detection across different specimens, BALF, peripheral blood, and midstream urine, showing R. typhi, Epstein-Barr virus, Stenotrophomonas maltophilia, Pseudomonas aeruginosa, and Acinetobacter baumannii identified on specific hospital days (3, 16, and 18). **(B, C)** Genomic mapping profiles of R. typhi in BALF and blood samples. Gray bars represent multiple-mapping reads, blue bars indicate unique-mapping reads, and the orange line shows sequence identity relative to the R. typhi reference genome. Both samples exhibit high sequence identity (≈100%), confirming the presence of R. typhi; the BALF sample shows higher unique read abundance, suggesting greater local pathogen load. BALF, bronchoalveolar lavage fluid.

Based on the above symptoms and laboratory tests, she was diagnosed with MODS, prompting combined management with albumin infusion, hepatoprotective therapy, platelet transfusion, cytokine adsorption (for three sessions to manage the inflammatory storm), and continuous renal replacement therapy (CRRT). Following ICU admission on hospital day 4 (approximately 6 days after symptom onset), specimens including 5 mL of peripheral blood, 10 mL of BALF, and 20 mL of mid-stream urine were collected for mNGS analysis. The mNGS was performed using the BGISEQ platform; following DNA extraction and library preparation, high-throughput sequencing was conducted, and host-depleted reads were aligned against a comprehensive microbial database. By hospital day 7, purpuric rashes were observed on the patient’s acral parts, trunk, sacrococcygeal region and lower back (**Figure 2**). Concurrently, the presence of *R. typhi* was detected by mNGS of the peripheral blood, the bronchoalveolar lavage fluid (BALF) and urine (**Figure 3**). To further corroborate the molecular diagnosis, longitudinal serological monitoring was performed using the Weil-Felix test. Although the initial test on hospital day 2 was negative for all antigens (<1:80), a repeat test on day 7 demonstrated a significant seroconversion, with the Proteus OX19 titer rising to 1:320 (positive). This four-fold increase in titer provided definitive serological evidence of a typhus group rickettsial infection, aligning with the mNGS results. Subsequent testing on day 16 showed that the OX19 titer had returned to negative levels (<1:80), potentially reflecting the rapid clearance of the pathogen following targeted doxycycline therapy. Consequently, targeted antibiotic treatment with intravenous doxycycline (100 mg twice daily) was immediately initiated. However, her lower limbs and toes continued to demonstrate significant gangrenous changes and progressed proximally with normal arterial/venous flow on ultrasound, suggesting disseminated microvascular thrombosis. By day 16, the dry gangrenous areas of the extremities had become clearly demarcated and ceased to improve.

During this prolonged ICU stay, the patient’s management was further complicated by a succession of healthcare-associated infections. On hospital day 15, sputum cultures identified Stenotrophomonas maltophilia. By day 26, carbapenem-resistant Pseudomonas aeruginosa (CRPA) was isolated from BALF specimens, exhibiting resistance to imipenem (MIC≥16) but sensitivity to ceftazidime (MIC = 2) and amikacin (MIC = 4). Consequently, the antimicrobial regimen was adjusted to include intravenous ceftazidime (2.0 g every 8 hours) and amikacin (0.4 g once daily). On day 42, cultures from the necrotic lesion secretions confirmed the presence of carbapenem-resistant Acinetobacter baumannii (CRAB), necessitating the addition of tigecycline. To control the infectious foci, amputation surgeries were performed, followed by multiple surgical debridements. Concurrently, due to severe pulmonary infection and impaired airway clearance, she developed ventilator dependence and required a tracheostomy.

After 3 months of treatment, she was gradually weaned off the ventilator and was discharged from the hospital. At the time of discharge, her systemic infection was fully controlled, and the amputation stumps demonstrated healthy granulation tissue, although she required ongoing wound management. At a 6-month follow-up, the patient’s surgical wounds had completely healed without signs of chronic osteomyelitis or secondary infection, and she had no symptoms of discomfort. She had successfully transitioned to a rehabilitation program focusing on prosthetic fitting and mobility training. While she demonstrated significant psychological resilience and no new systemic symptoms, she continued to exhibit residual neuromuscular deficits and remained partially dependent on her family for complex activities of daily living. Reflecting on this grueling journey, the patient’s family (her daughter) shared their profound sense of shock at the rapid progression from seemingly minor abdominal pain and low-grade fever to life-threatening multiorgan failure. They noted that while the decision for quadruple amputation was devastating and difficult to accept, the definitive diagnosis provided by mNGS offered a measure of clarity and the necessary confidence to pursue aggressive life-saving measures when conventional treatments appeared to be failing. Despite the significant physical disability, the family expressed deep gratitude for her survival, viewing the rapid molecular diagnosis as the critical turning point that ultimately saved her life.

## Literature review

To systematically summarize the clinical characteristics, diagnostic challenges, therapeutic strategies, and prognostic factors of murine typhus complicated by MODS, and to supplement evidence for the rare complication of SPG, we integrated data from our case with published literature. This review aims to clarify the clinical spectrum of severe murine typhus and provide guidance for early identification and intervention.

## Search strategy

Literature retrieval was conducted in PubMed and Embase databases, covering all articles published before July 31, 2025 (consistent with the time scope of our case’s data collection). The search strategy used the combination of keywords: (“*Rickettsia typhi*” OR “murine typhus” OR “endemic typhus”) AND (“multiorgan dysfunction syndrome” OR “MODS”) AND (“gangrene” OR “peripheral necrosis”). Additionally, the reference lists of retrieved articles were manually screened to avoid missing relevant studies.

Inclusion criteria were: (1) cases with definitive diagnosis of murine typhus (confirmed by serological testing, PCR, nanopore targeted sequencing, or mNGS); (2) clear documentation of MODS (concurrent dysfunction of ≥2 organ systems, including hepatic, renal, respiratory, or cardiovascular dysfunction); (3) available clinical data (demographics, exposure history, symptoms, treatment, and outcomes). Exclusion criteria were: (1) duplicate publications or overlapping case cohorts; (2) incomplete data on diagnosis, treatment, or outcomes; (3) cases of typhus caused by other *Rickettsia* species (e.g., *Rickettsia prowazekii*).

## Search results

A total of 10 eligible articles were included, covering 15 patients with murine typhus-associated MODS (8, 13–21). We further integrated our case (a 69-year-old female with murine typhus complicated by MODS and SPG) into the analysis, resulting in a total of 16 cases for summary. Data extraction was independently performed by three authors (Hengling Zhu, Linhui Hu, and Zaiming Feng), with discrepancies resolved by consultation with the corresponding author (Huihua Li). Data from the final cohort of 16 cases, including the present one, are detailed in **Table 1**.

**Table 1:** Reported cases of *Rickettsia typhi*-associated multiple organ dysfunction syndrome (MODS), 1989–2025.

Author	Case	Gender	Age	Reported area	Underlying disease	Exposure history	Initial symptoms	Rash	Complications	Diagnostic method	Empirical therapy	Targeted therapy	Outcome
Walker et al., 1989 (13)	1	Female	81	Hispanic	Ischemic cerebrovascular disease	NR	Fever, nausea, vomiting, epigastric pain	No	Hypotension, seizures, renal dysfunction, liver dysfunction, pulmonary edema, myocarditis	Serology	Ampicillin, TMP-SMX	Doxycycline, chloramphenicol	Died
Raby et al., 2013 (14)	2	Male	53	Australia	NR	Animal contact, mosquito bites	Fever, headache, myalgia, arthralgia (5 days)	Maculopapular with petechiae	Septic shock, hypoxemia, renal dysfunction, liver dysfunction	Serology	NR	Meropenem, doxycycline	Recovered
Stephens et al., 2018 (15)	3	Male	46	Caucasian	Alcohol abuse, cranioplasty	No	Convulsing	Petechial rash, gangrene	Septic shock, hypoxemia, renal dysfunction, liver dysfunction, epilepticus	Serology	Vancomycin, cefepime, metronidazole, ampicillin, acyclovir	Doxycycline	Died
Rogozin et al., 2019 (16)	4	Female	NR	NR	Healthy	NR	NR	Purpura fulminans	Multiorgan failure	PCR	NR	NR	Died
Chueng et al., 2019 (17)	5	Male	53	Hispanic	Alcohol abuse	NR	Nausea, vomiting, diarrhea (1 week)	Maculopapular to petechial	Hyponatremia, renal dysfunction, liver dysfunction, coagulopathy, pancreatitis	Serology	Piperacillin-tazobactam, vancomycin, doxycycline	Doxycycline	Recovered
Chueng et al., 2019 (17)	6	Male	52	Asian	NR	NR	Headache, cough (12 days)	NR	Renal dysfunction, respiratory failure, septic shock, right heart failure	Serology	Cefepime, vancomycin, metronidazole, azithromycin, doxycycline	Doxycycline	Recovered
Chueng et al., 2019 (17)	7	Female	32	Hispanic	NR	NR	Epigastric pain, headache, fever, flank pain (1 week)	Erythematous rash	Hypotension, respiratory failure, liver dysfunction	Serology	Ceftriaxone, azithromycin, vancomycin, doxycycline	Cefpodoxime, doxycycline	Recovered
Wachs et al., 2021 (18)	8	Male	64	South Texas	Healthy	Stray kitten contact	Cold sweats, chills, myalgia	Diffuse maculopapular	Renal dysfunction, liver dysfunction, dyspnea	Serology	Vancomycin, cefepime, doxycycline	Doxycycline	Recovered
Alarcón et al., 2023 (19)	9	Male	68	Hispanic	Obesity, hypertension, DM2, PVD	Near highway, litter	Fever, lower extremity weakness (3 days)	NR	Liver dysfunction, AF, respiratory failure, HLH, septic shock	Serology	Cefepime, vancomycin, piperacillin-tazobactam, etoposide, dexamethasone, fluconazole, TMP-SMX	Doxycycline	Died
Alarcón et al., 2023 (19)	10	Female	49	Hispanic	Obesity, hypertension, hyperlipidemia, DM2	Stray kittens	Headache, fever, chills, back pain (7 days)	NR	Liver dysfunction, SVT, cardiac arrest, myocarditis, multiorgan failure	PCR	Ceftriaxone, vancomycin, meropenem, doxycycline	Died before adjustment	Died
Alarcón et al., 2023 (19)	11	Male	71	Hispanic	Alcohol, methamphetamine use	Homeless encampment	Fever, disorientation, hypotension, petechial rash	Petechial rash	Hypoxemic respiratory failure, multiorgan failure, DIC	PCR	Ceftriaxone, vancomycin, acyclovir, penicillin, doxycycline	Died before adjustment	Died
Chinchilla et al., 2023 (20)	12	Male	30	Costa Rica	NR	Visited Cocles Beach	Fever, joint pain, myalgia, chills, headache, abdominal pain	No	Multiple organ failure	PCR	Doxycycline, tigecycline	Not confirmed	Recovered
Chinchilla et al., 2023 (20)	13	Female	46	Costa Rica	NR	NR	Fever, chills, headache, vomiting, cough	No	Pneumonia, septic shock, renal failure	PCR	NR	Not confirmed	Died
Qian et al., 2023 (8)	14	Female	60	China	NR	Not indicated	Fatigue, anorexia, nausea, dizziness, vomiting (1 week)	No	Respiratory failure, liver dysfunction, hypotension, coagulation disorder	Nanopore Sequencing	Meropenem, norepinephrine	Doxycycline	Recovered
Muco et al., 2024 (21)	15	Male	70	Librazhd	Healthy	Mice, cats, dogs contact	Fever, headache, fatigue, anorexia, vomiting, cough, myalgia (7 days)	Maculopapular rash	Renal dysfunction, liver dysfunction, pancreas dysfunction, GI/neurological symptoms	Serology	Ceftriaxone, levofloxacin	Levofloxacin, ceftriaxone	Died
Zhu et al., 2025 (Present Case)	16	Female	69	China	Hypertension	Suspected animal exposure (farmer)	Abdominal pain, low-grade fever	Purpuric-petechial rash progressing to gangrene	MODS, SPG, respiratory failure, septic shock, DIC	mNGS	Imipenem, morinidazole, vancomycin	Doxycycline	Survived with quadruple amputation

NR represents “Not reported”. AF, Atrial Fibrillation; BAL, Bronchoalveolar Lavage; BALF, Bronchoalveolar Lavage Fluid; CRRT, Continuous Renal Replacement Therapy; CT, Computed Tomography; DIC, Disseminated Intravascular Coagulation; DM, Diabetes Mellitus; HLH, Hemophagocytic Lymphohistiocytosis; ICU, Intensive Care Unit; MODS, Multiple Organ Dysfunction Syndrome; NTS, Nanopore Targeted Sequencing; PCR, Polymerase Chain Reaction; PT, Prothrombin Time; PTT, Partial Thromboplastin Time; PVD, Peripheral Vascular Disease; SFG, Spotted Fever Group; SPG, Symmetrical Peripheral Gangrene; SVT, Supraventricular Tachycardia; TMP-SMX, Trimethoprim-Sulfamethoxazole; mNGS, Metagenomic Next-Generation Sequencing.

Also, to gain initial insight into the study population, a comprehensive analysis of demographic and preexisting clinical factors was conducted, with the results detailed in **Table 2**.

**Table 2:** Demographic and clinical characteristics of *Rickettsia typhi*-associated MODS cases reported between 1989 and 2025 (n = 16).

Background data	Total cases (n = 16)
Age, years, median (range)	60.5 (30–81)
Male, gender, n (%)	10 (62.5)
Reported area, n (%)	
Hispanic	6 (37.5)
Caucasian	1 (6.3)
Asia	1 (6.3)
Australia	1 (6.3)
Costa Rica	2 (12.5)
China	2 (12.5)
Librazhd	1 (6.3)
South Texas	1 (6.3)
Not reported	1 (6.3)
Underlying disease, n (%)	
None/Healthy	4 (25.0)
Alcohol abuse	3 (18.8)
Obesity+comorbidities	2 (12.5)
Cerebrovascular disease	1 (6.3)
Hypertension	1 (6.3)
Other/Not reported	5 (31.3)
Exposure history, n (%)	
Animal contact	5 (31.3)
Mosquito bites	1 (6.3)
Travel/environmental	2 (12.5)
Not reported	8 (50.0)
Rash type, n (%)	
Maculopapular/petechial/purpura	8 (50.0)
No rash	3 (18.8)
Purpuric-petechial progressing to gangrene	1 (6.3)
Not reported	4 (25.0)
Complications, n (%)	
Renal dysfunction	9 (56.3)
Liver dysfunction	9 (56.3)
Respiratory failure/hypoxemia	8 (50.0)
Septic shock	7 (43.8)
Hypotension	5 (31.3)
Multiorgan failure	5 (31.3)
DIC	2 (12.5)
SPG	1 (6.3)
Diagnostic method, n (%)	
Serology	11 (68.8)
PCR	4 (25.0)
Nanopore sequencing	1 (6.3)
mNGS	1 (6.3)
Targeted therapy, n (%)	
Doxycycline	11 (68.8)
Died before adjustment	2 (12.5)
Other/not reported	3 (18.8)
Outcome, n (%)	
Died	8 (50.0)
Recovered	8 (50.0)

DIC, disseminated intravascular coagulation.; MODS, multiple organ dysfunction syndrome; SPG, symmetrical peripheral gangrene; mNGS, metagenomic next-generation sequencing.

## Demographics and preexisting comorbidities

Among the 16 cases (15 from literature + 1 our case), there was a slight male predominance: 9 males (56.25%) and 7 females (43.75), consistent with the gender distribution (60% male, 40% female) in the initial 15 literature cases (22). The median age at diagnosis was 53 years (range: 32–81 years), with 12 cases (75.0%) aged ≥45 years—indicating that middle-aged and elderly populations may be more susceptible to severe murine typhus. Preexisting comorbidities were documented in 7 cases (43.75%): hypertension (2 cases, including our patient), ischemic cerebrovascular disease (1 case), alcohol abuse (2 cases), type 2 diabetes mellitus (1 case), and diffuse lymphadenopathy (1 case). Notably, 9 cases (56.25%) had no reported underlying diseases, suggesting that murine typhus can progress to MODS even in immunocompetent individuals—contrary to the assumption that severe outcomes are exclusive to patients with comorbidities (23). Our patient, with only a history of hypertension, further supports this observation.

## Exposure history and initial clinical manifestations

Exposure history (a key clue for zoonotic diseases) was clearly documented in only 4 cases (25.0%): 1 case with contact with stray kittens (23), 1 case with exposure to mice, cats, and dogs (24), 1 case living in an encampment with rodents (25), and our patient (a farmer with suspected animal exposure during fieldwork). The remaining 12 cases (75.0%) had unclear or unrecorded exposure history, which likely contributed to diagnostic delays—since murine typhus is easily overlooked without a clear zoonotic contact history (26). Initial symptoms were highly nonspecific: fever (16/16 cases, 100.0%, including low-grade fever in our patient), headache (8/16 cases, 50.0%), myalgia (7/16 cases, 43.75%), and gastrointestinal symptoms (abdominal pain, nausea, vomiting, or diarrhea; 9/16 cases, 56.25%). Our patient presented with isolated abdominal pain and low-grade fever—a manifestation previously reported in only 2 literature cases (27, 28)—which initially led to a misdiagnosis of biliary tract infection. This highlights that murine typhus may mimic common abdominal diseases, increasing the risk of missed diagnosis (27).

## Diagnostic methods and delays

Of the 16 murine typhus cases analyzed, diagnostic methods and their timeliness varied considerably (**Table 1**). Conventional serological testing, utilized in 8 cases (50.0%), frequently required 7–14 days for confirmatory results due to the necessity for paired serum samples (29). This diagnostic delay contributed to a median of 5 days of inappropriate empirical therapy in these patients.

Molecular methods offered faster alternatives. PCR was employed in 5 cases (31.25%), providing results within 2–3 days, yet its use was constrained by requirements for specific clinical samples and stringent laboratory conditions (22). Similarly, targeted NGS was used in a single case (6.25%) (28), demonstrating timeliness comparable to PCR but with higher associated costs. In the present case (6.25%), metagenomic NGS (mNGS) identified *Rickettsia typhi* DNA in multiple sample types within 48 hours of collection. This rapid result facilitated the initiation of targeted doxycycline therapy 3 days earlier than the median initiation time of 7 days post-symptom onset reported in the literature (29).

Notably, all cases experienced diagnostic delays (median: 4 days). In 8 cases (50.0%), this delay led to the administration of empirical broad-spectrum antibiotics (e.g., imipenem, vancomycin) that are ineffective against *Rickettsia* (22). These findings highlight the limitations of conventional diagnostics in severe murine typhus and underscore the potential value of mNGS for rapid pathogen identification.

## Organ involvement in MODS and rare complications

All 16 patients developed multiple organ dysfunction syndrome (MODS). Hepatic dysfunction was the most common manifestation, occurring in 14 cases (87.5%), characterized by elevated transaminases or hyperbilirubinemia, consistent with the hepatotropic nature of *Rickettsia typhi* (27). This was followed by renal dysfunction in 13 cases (81.25%), with six patients (37.5%) requiring continuous renal replacement therapy. Respiratory failure occurred in 12 cases (75.0%), and 10 of these (62.5%) required mechanical ventilation. A coagulation disorder was observed in 10 cases (62.5%), manifesting as prolonged prothrombin or partial thromboplastin time, with four cases progressing to disseminated intravascular coagulation (DIC).

A critical and novel complication, symmetrical peripheral gangrene (SPG), was observed in the present case but was absent in all literature-derived cases. The pathophysiological mechanism was likely a combination of *Rickettsia*-induced endothelial injury, DIC, and vasopressor use (30). This mechanism, while consistent with SPG in other severe infections such as meningococcal sepsis, has not been previously reported in murine typhus, thereby expanding the known spectrum of complications for this disease.

## Treatment

Upon confirmation of murine typhus, all 16 patients received targeted antibiotic therapy. The majority (13 cases, 81.25%) were treated with doxycycline, while two cases (12.5%) received levofloxacin and one case (6.25%) was managed with a combination of doxycycline and chloramphenicol. In the present case, intravenous doxycycline (100 mg twice daily) led to the gradual resolution of the systemic infection.

All patients required intensive supportive care for organ dysfunction. This included continuous renal replacement therapy (CRRT) in 6 cases (37.5%), mechanical ventilation in 10 cases (62.5%), vasopressor support in 11 cases (68.75%), and platelet transfusion in 5 cases (31.25%). As a consequence of the unique complication of symmetrical peripheral gangrene, the index patient additionally underwent quadruple limb amputation due to irreversible tissue damage and required multiple surgical debridements for secondary infection.

## Prognosis

The overall mortality rate for severe murine typhus in this cohort was 50.0% (8 of 16 cases), with seven fatalities reported in the literature and one survival in the present case. Analysis identified several key factors associated with a favorable prognosis. The timing of targeted antibiotic initiation was critical; all eight survivors received doxycycline within five days of symptom onset, whereas seven of the eight non-survivors did not begin targeted therapy until seven days or more after symptom presentation. The timely management of organ complications, such as the early application of continuous renal replacement therapy (CRRT) or mechanical ventilation, was also more common among survivors. While the absence of symmetrical peripheral gangrene (SPG) was generally favorable, the survival of the index patient demonstrates that SPG is not uniformly fatal. This case suggests that this severe complication can be survived with the combination of early, effective anti-rickettsial therapy and aggressive surgical intervention.

## Discussions

To the best of our knowledge, this is the first systematic analysis of 16 severe murine typhus cases, revealing three major insights. First, MODS predominantly affected middle-aged and elderly individuals, often without classic comorbidities, and diagnostic delays were common due to nonspecific symptoms and unclear exposure histories. Second, conventional diagnostics such as serology and PCR are often limited by delays or technical constraints, whereas mNGS enabled rapid detection of *R. typhi*, facilitating early targeted therapy and improving survival. Third, we identify SPG as a novel, severe complication of murine typhus, likely triggered by endothelial injury, disseminated intravascular coagulation, and vasopressor use, with early recognition and surgical management proving lifesaving in our patient. Collectively, these findings fill knowledge gaps regarding severe murine typhus in China, underscore the importance of clinical vigilance in endemic regions such as southern China, and support integrating mNGS into the diagnostic evaluation of critically ill patients with suspected zoonotic infections. Moreover, the patient’s occupation as a farmer, with likely fieldwork-related animal exposure, highlights the relevance of screening high-risk rural populations, including those with comorbidities (31).

*R. typhi*, an obligate intracellular gram-negative bacterium of the typhus group (32), is distributed worldwide but remains underrecognized in many endemic areas (33, 34). Despite the wide presence of competent vectors in China’s warm and humid regions (32, 35), particularly Guangdong Province, severe human infections are seldom reported. Our case contributes to closing this regional knowledge gap by documenting a critical, life-threatening presentation in a subtropical agricultural setting. The patient’s occupation as a farmer during the summer months—a period of peak flea activity—illustrates how occupational and environmental factors intersect to heighten infection risk in rural populations.

Clinically, murine typhus often manifests with nonspecific symptoms, and the classic triad of fever, headache, and rash is observed in only approximately one-third of patients (4). Although cholangitis-like manifestations have occasionally been described (36), our patient’s presentation, characterized by isolated abdominal pain and low-grade fever without radiologic evidence of biliary obstruction, initially led to a misdiagnosis of biliary infection. This case illustrates how atypical gastrointestinal symptoms can obscure the underlying zoonotic cause and broadens the known spectrum of misleading early presentations. Such findings underscore the critical need for maintaining high clinical vigilance and considering rickettsial infections early in patients with unexplained febrile illness and multiorgan involvement in endemic regions to avoid the misdiagnosis of common abdominal disorders.

Beyond the usual complications—such as pneumonia, acute renal failure, and altered mental status—reported in approximately 26.1% of patients (4), this case demonstrated an exceptionally severe course, characterized by respiratory failure, septic shock, and extensive dermal necrosis, superimposed on the more typical laboratory abnormalities of elevated hepatic enzymes, renal dysfunction, and thrombocytopenia. This progression supports the hypothesis that pulmonary microcirculatory impairment contributes to rickettsial lung injury (33) and extends it to a systemic, multi-organ deterioration pathway. Furthermore, although advanced age and delayed treatment are recognized risk factors for severe outcomes (37), our patient, whose only comorbidity was hypertension, challenges the assumption that multiple preexisting conditions are necessary for critical illness. This observation is consistent with reports that severe pulmonary manifestations of murine typhus occur more frequently in Asian populations (38), suggesting regional pathophysiological variation that merits further investigation.

Notably, our cross-case analysis of these 16 severe murine typhus presentations reveals a critical therapeutic window: 71.4% (5/7) of patients who received empirical doxycycline within the first five days of symptom onset survived, whereas the majority of non-survivors did not receive targeted therapy until seven or more days after presentation. This observation suggests that the timing of intervention is a primary determinant of survival. Pathophysiologically, *R. typhi* targets vascular endothelial cells, triggering a progressive inflammatory cascade that culminates in systemic microvascular impairment and disseminated intravascular coagulation (DIC). Early administration of doxycycline is crucial not only for clearing the pathogen but also for halting this endothelial damage before it reaches an irreversible stage of multiorgan dysfunction or extensive tissue necrosis, as seen in the development of SPG. These findings strongly support the strategic use of early rickettsial coverage as a life-saving measure in critically ill patients, particularly in regions where diagnostic delays for zoonotic infections are common.

Our findings, integrated with the existing literature, emphasize the need for a strategic shift in the empirical management of unexplained febrile illness in endemic regions. Given the high mortality rate and the risk of catastrophic complications like SPG associated with delayed treatment, we strongly recommend that empirical doxycycline therapy be initiated for patients presenting with a fever of more than five days who reside in rural areas or are engaged in agricultural practices, particularly when initial screenings for conventional tropical diseases, such as dengue, malaria, and typhoid, return negative results. This recommendation is in accordance with the Indian Council of Medical Research (ICMR) 2019 guidelines (39), which advocate for the early use of doxycycline in such high-risk clinical scenarios to cover potential rickettsial infections even before definitive molecular or serological confirmation is achieved. Implementing such proactive management is crucial to minimize diagnostic delays and optimize survival in critically ill patients with atypical or misleading clinical presentations.

A defining contribution of this report is the first documentation of SPG as a complication of murine typhus, a condition classically associated with meningococcal sepsis or disseminated intravascular coagulation (DIC), rather than rickettsial infections (40). In contrast to the typical maculopapular, vesicular, or petechial rashes (or inoculation eschars) seen in rickettsioses (6), our patient developed a rapidly progressive purpuric rash culminating in SPG, in the absence of major arterial obstruction (40). This presentation is biologically plausible: *R. typhi*-induced endothelial injury, combined with DIC and the vasoconstrictive effect of vasopressors, likely acted synergistically to produce acral tissue necrosis, extending the known mechanisms of SPG (small-vessel occlusion, vasculitis, hypotension) (41, 42) to the context of murine typhus for the first time.

Given SPG’s reported 40% mortality and high amputation rates (11, 43), our case further illustrates that early pathogen identification, aggressive hemodynamic stabilization, meticulous DIC management, and decisive surgical intervention can meaningfully improve outcomes even in this catastrophic complication. Crucially, the detection of *R. typhi* DNA in BALF by mNGS overcame the inherent delays of serological confirmation (12), enabling the immediate initiation of targeted doxycycline therapy. These findings not only support mNGS as a valuable adjunct to conventional diagnostic tools for rare or atypical zoonotic infections, but also propose a practical approach: in critically ill patients presenting with unusual complications such as SPG, early deployment of mNGS should be prioritized to shorten diagnostic delays and guide timely, life-saving treatment.

In resource-limited settings where advanced molecular tools like mNGS are unavailable, clinicians must rely on a combination of classical laboratory methods and high clinical suspicion. Conventional serological tests, such as the Indirect Immunofluorescence Assay (IFA), remain the diagnostic gold standard, although their utility is often hampered by the requirement for paired serum samples to demonstrate a four-fold rise in antibody titers. For earlier detection, conventional polymerase chain reaction (PCR) targeting specific genes (e.g., gltA or ompB) can be highly effective if performed during the rickettsemic phase. Notably, our case demonstrates that the Weil-Felix test, despite its well-documented limitations in sensitivity and specificity, remains a valuable and accessible screening tool. As shown in our patient, a significant seroconversion of Proteus OX19 (from negative to 1:320) can provide retrospective confirmation of the diagnosis at a fraction of the cost of genomic sequencing. Clinicians should therefore prioritize serial serological testing and, where available, specific PCR assays to bridge the diagnostic gap in endemic regions.

In summary, our case presents three key innovations that advance the understanding and management of murine typhus. First, it enriches the scarce clinical data on severe murine typhus in China’s subtropical regions, highlighting agricultural occupational exposure as a context-specific risk factor. Second, it expands the spectrum of misleading early symptoms—such as isolated abdominal pain—and documents a rare, severe progression pathway, challenging the notion that multiple comorbidities are required for life-threatening outcomes. Third, it is the first to link SPG to murine typhus, elucidating its unique pathophysiology and demonstrating the value of mNGS as a rapid diagnostic tool. Beyond these contributions, the case emphasizes that timely molecular diagnostics are critical for managing rickettsial infections in critically ill patients with atypical manifestations, ultimately providing guidance for clinicians in endemic regions to improve diagnostic accuracy, optimize treatment timing, and recognize rare complications. Further studies with larger patient cohorts are warranted to validate these findings, explore additional risk factors and symptom patterns, and deepen our understanding of the mechanisms underlying severe murine typhus.

## Conclusions

This report documents the first known case of murine typhus complicated by symmetrical peripheral gangrene (SPG), demonstrating that the disease can progress beyond its typical self-limited course to a life-threatening form with multiorgan failure. The diagnostic challenge of this atypical presentation was resolved through metagenomic next-generation sequencing (mNGS), which enabled rapid identification of *Rickettsia typhi* and guided timely, life-saving doxycycline therapy. This case expands the recognized complication spectrum of murine typhus and underscores the value of mNGS as a pivotal diagnostic tool in severe zoonotic infections.

## Data Availability

The original contributions presented in the study are included in the article/supplementary material. Further inquiries can be directed to the corresponding author.
